# Correction: Post-COVID-19 syndrome: Physical capacity, fatigue and quality of life

**DOI:** 10.1371/journal.pone.0320612

**Published:** 2025-03-20

**Authors:** The PLOS One Staff

## Notice of republication

The third author’s name should be Meike Dirks not Dirks Meike.

## Supporting information

S1 File Originally published, uncorrected article. (PDF)

S2 File Republished, corrected article. (PDF)
