# Supplementary material for: Correction: Post-COVID-19 syndrome: Physical capacity, fatigue and quality of life
Source: PLoS One. 2025 Mar 20;20(3):e0320612. doi: 10.1371/journal.pone.0320612 (PMC11925273; doi:10.1371/journal.pone.0320612)
Supplement: S1 File — (PDF) [file pone.0320612.s001.pdf]

PLOS ONE

RESEARCH ARTICLE

Post-COVID-19 syndrome: Physical capacity, fatigue and quality of life

Sebastian Beyer[1☯*], Sven Haufe[1☯], Meike Dirks[2], Michèle Scharbau[3], Viktoria Lampe[1], Alexandra Dopfer-Jablonka[4], Uwe Tegtbur[1], Isabell Pink[3], Nora Drick[3☯], Arno Kerling[1☯]

1 Department of Rehabilitation and Sports Medicine, Hannover Medical School, Hannover, Germany, **2** Clinic for Neurology, Hannover Medical School, Hannover, Germany, **3** Department of Respiratory Medicine, Hannover Medical School, Hannover, Germany, **4** Clinic for Rheumatology and Immunology, Hannover Medical School, Hannover, Germany

☯ These authors contributed equally to this work.
* beyer.sebastian@mh-hannover.de

Check for updates

OPEN ACCESS

Citation: Beyer S, Haufe S, Dirks M, Scharbau M, Lampe V, Dopfer-Jablonka A, et al. (2023) Post-COVID-19 syndrome: Physical capacity, fatigue and quality of life. PLoS ONE 18(10): e0292928. https://doi.org/10.1371/journal.pone.0292928

Editor: Kalyana Chakravarthy Bairapareddy, University of Sharjah, UNITED ARAB EMIRATES

Received: July 5, 2023

Accepted: October 3, 2023

Published: October 23, 2023

Peer Review History: PLOS recognizes the benefits of transparency in the peer review process; therefore, we enable the publication of all of the content of peer review and author responses alongside final, published articles. The editorial history of this article is available here: https://doi.org/10.1371/journal.pone.0292928

Copyright: © 2023 Beyer et al. This is an open access article distributed under the terms of the Creative Commons Attribution License, which permits unrestricted use, distribution, and reproduction in any medium, provided the original author and source are credited.

Data Availability Statement: You can access the study data through the following links: https://mhh-publikationsserver.gbv.de/receive/mhh_mods_00002583 or https://doi.org/10.26068/mhhrpm/20231010-000.

Abstract

Purpose

Post-Covid-19 syndrome is defined as the persistence of symptoms beyond 3 months after severe acute respiratory syndrome coronavirus 2 (SARS-CoV-2) infection. The most common symptoms include reduced exercise tolerance and capacity, fatigue, neurocognitive problems, muscle pain and dyspnea. The aim of our work was to investigate exercise capacity and markers of subjective wellbeing and their independent relation to post-COVID-19 syndrome.

Patients and methods

We examined a total of 69 patients with post-COVID-19 syndrome (23 male/46 female; age 46±12 years; BMI 28.9±6.6 kg/m^2) with fatigue and a score ≥22 in the Fatigue Assessment Scale (FAS). We assessed exercise capacity on a cycle ergometer, a 6-minute walk test, the extent of fatigue (FAS), markers of health-related quality of life (SF-36 questionnaire) and mental health (HADS).

Results

On average the Fatigue Assessment Scale was 35.0±7.4 points. Compared with normative values the VO2max/kg was reduced by 8.6±5.8 ml/min/kg (27.7%), the 6MWT by 71±96 m (11.9%), the health-related quality of life physical component score by 15.0±9.0 points (29.9%) and the mental component score by 10.6±12.8 points (20.6%). Subdivided into mild fatigue (FAS score 22–34) and severe fatigue (FAS score ≥35), patients with severe fatigue showed a significant reduction of the 6-minute walk test by 64±165 m ($p<0.01$) and the health-related quality of life physical component score by 5.8±17.2 points ($p = 0.01$). In multiple regression analysis age ($\beta = -0.24$, $p = 0.02$), sex ($\beta = 0.22$, $p = 0.03$), mental ($\beta = -0.51$, $p<0.01$) and physical ($\beta = -0.44$, $p<0.01$) health-related quality of life and by trend the 6-minute walk test ($\beta = -0.22$, $p = 0.07$) were associated with the FAS.

Funding: YES - The study was supported and funded by Erwin Röver Foundation. The funders had no role in study design, data collection and analysis, decision to publish, or preparation of the manuscript.

Competing interests: NO authors have competing interest.

Conclusion

Patients with post-COVID-19 syndrome show reduced maximal and submaximal physical performance as well as limitations in quality of life, particularly pronounced in the physical components. These results are essentially influenced by the severity of fatigue and implicating the need for targeted treatments.

Introduction

The post-acute sequelae of severe acute respiratory syndrome coronavirus 2 (SARS-CoV-2) infection—also referred to as post-COVID-19 –is estimated to be prevalent in at least 65 million individuals worldwide [1]. According to the WHO post-COVID-19 syndrome (PCS) is defined as the persistence of symptoms beyond 3 months after severe acute respiratory syndrome coronavirus 2 (SARS-CoV-2) infection [2]. Approximately 80% of hospitalized and non-hospitalized patients have symptoms lasting longer than 12 months [3]. Important risk factors associated with PCS appears to be pre-existing illnesses (especially cardiorespiratory, autoimmune and oncological previous diseases, and neuropsychiatric disorders), female gender, respiratory symptoms at onset, length of hospital stay and severity of illness, although milder courses also can trigger PCS [1, 4–6]. The most common symptoms that contribute mainly to decreased health recovery and reduced resilience include fatigue, reduced exercise tolerance and muscular strength, neurocognitive problems, muscle pain and dyspnea [7, 8].

Physical activity and exercise capacity are significant predictors of long-term survival and both are reduced in the context of PCS [9–12]. In contrast good physical performance to a certain extent protects against a severe course of SARS-CoV-2 infection and reduces the likelihood of hospitalization [8, 13]. In addition, there is a strong relation between physical performance and health-related quality of life (HrQoL), and exercise training is used as a therapeutic tool for a variety of physical and mental disorders [14–16]. Mental and physical components of HrQoL are reduced after acute SARS-CoV-2 infection as well as in PCS patients [17], both by the disease itself and by public health measures such as quarantine [18, 19].

However, the relative negative impact of PCS on physical function, fatigue, mental conditions and their associations are not fully elucidated. This knowledge is vital for a targeted treatment because until now a specific causal therapy for PCS patients does not exist. We aimed to identify potential predictors that contribute to PCS-related fatigue and thereby might help to better understand the relative significance of physical and mental risk factors on their association with PCS severity. We conducted a thorough assessment of patients after SARS-CoV-2 infection and diagnosed PCS and analyzed associations of fatigue severity with parameters of physical capabilities, quality of life and mental conditions in order to be able to implement an optimal therapy strategy in the long term.

Methods

Study design and participants

The current evaluation was a cross-sectional analysis of data collected between September 2021 and June 2022. The study was conducted at a university medical hospital in the city of Hannover (Lower Saxony, Germany) with individuals diagnosed with PCS. We included patients from October 2021 to May 2022 who consulted the pneumological post-COVID-19 outpatient clinic of Hannover Medical School, which is the first point of contact for almost all patients with suspected post-COVID19 syndrome, regardless of the various possible symptoms

that could lead to the diagnosis of PCS. Patients were referred to our university hospital by general practitioners or pneumologists due to persisting symptoms ≥3 months after SARS--CoV-2 infection.

According to pre-study defined criteria, we included female and male volunteers aged 18 years or older who reported a 3-month continuing impairment of capability after COVID-19 (detection by PCR) infection with a Fatigue Assessment Scale (FAS) score ≥22 points. Exclusion criteria were current participation in another intervention study, clinically relevant acute or chronic infections, pregnancy, preceding surgery less than 8 weeks ago, joint replacement that is less than 6 months old, tumor -associated diseases in the last 5 years such as any illnesses or functional impairments which preclude participation in a physical training intervention.

The Clinic for Rehabilitation and Sports Medicine at Hannover Medical School in cooperation with the Department of Respiratory Medicine was responsible for the study design, statistical planning, inclusion of study participants, data collection and analysis, as well as the preparation of the manuscript. This study was carried out in accordance with the Declaration of Helsinki and is registered on German Clinical Trials Register (registration number: DRKS0002793). The institutional ethics review board of Hannover Medical School approved the study (No.9822_BO_S_2021) and written informed consent was obtained prior to the inclusion of participants.

Assessments

After study inclusion, all subjects completed a comprehensive medical evaluation including pulmonary function testing measured by body plethysmography standardized to European Respiratory Society (ERS). We assessed height and weight in a standardized fashion and estimated fat and fat-free mass with a bioimpedance analysis (InBody 720, Biospace, Seoul, Korea). To determine steps per day, patients received a wearable (Forerunner 45, Garmin, Olathe, United States).

Maximal and submaximal exercise testing

For testing parameters of exercise capacity and maximum power output patients performed an incremental exercise test using a spirometric system (Oxycon CPX, CareFusion, Würzburg, Germany) on a speed independent bicycle ergometer (Ergoline P150, Bitz, Germany) with 60 to 70 revolutions per minute. The exercise test were carried out in the presence of a physician (specialist for cardiology or internal medicine) together with a sports scientist or a medical technical assistant, all experienced and trained in conducting, evaluating and interpreting exercise performance diagnostics. Except six examinations the incremental test started with a load of 20 Watt (W) increasing in 10 W steps every minute and was stopped with the onset of subjective overexertion because of peripheral muscle fatigue and/or pulmonary limitations (four started with a load of 50 W increasing in 10 W steps every minute, two started with a load of 50 W increasing in 16.67 W steps every minute). The subjective perceived exertion was assessed by the Borg-scale [20]. Heart rate and oxygen uptake were continuously measured breath by breath. Body weight normalized values for maximum power output and VO_2max were also expressed as percentage to age- and gender-adjusted reference values [21, 22].

The 6MWT is a test to assess submaximal exercise capacity by walking distance. Participants walked a slope-free corridor for a total of six minutes at their own speed and the reached distance was recorded in meters [23]. The tests were supervised by a trained sports scientists or medical technical assistants.

Questionnaires

We distributed a questionnaire for the estimation of HrQoL (short form 36 [SF-36]). The SF-36 using eight subscales, each with a scale ranging from 0 to 100, culminating in two summated scales, the mental and physical component score. A higher mental and physical component score means a better HrQol. In addition, we calculated the mean of the eight subscales for classification of our patients in comparison with a systematic review [24].

We assessed the severity of depression and anxiety with the Hospital Anxiety and Depression Scale (HADS) [25]. Scores for the anxiety and depression subscales range from 0 to 21, higher scores indicating more severe anxiety or depression [25]. Fatigue was measured with the FAS (© FAS Fatigue Assessment Scale: ild care foundation [http://www.ildcare.nl]). We divided the patients into two groups, the subdivision between fatigue (n = 32) and extreme fatigue (n = 37) is a FAS score $\geq$35 points.

Statistical analysis

Data were first tested for normal distribution and variance homogeneity with Kolmogorov-Smirnov test and the Levene test, respectively. Differences between baseline parameters were compared by Student t test for unpaired samples, the Mann-Whitney u test or the chi-square test, respectively. Univariate associations between parameters were tested using Pearson's correlation coefficient or Spearman's correlation coefficient. Differences between subgroups of fatigue and extreme fatigue (based on FAS score) were analyzed by Student t test for unpaired samples or the Mann-Whitney u test. Differences between subgroups regarding vaccination status were tested with a one-way ANOVA and Bonferroni´s *post hoc* test.

Finally, a stepwise backward multivariate linear regression was performed to identify parameters associated with PCS severity. The type-I-error was set to 5% (two-sided). All statistical analyses were performed with IBM SPSS 27 Statistics (IBM Corporation, New York, USA). Unless otherwise stated, values are given as mean ± SD or as median [min; max].

Results

Between September 2021 and June 2022, 77 patients were recruited for study examinations. Of those, eight have to be excluded as they did not meet the in-/ exclusion criteria. Four withdrew their consent to participate in the study, one did not appear for examinations, one was too young, one was unable to confirm a positive PCR/antigen test, and one had a FAS score of less than 22 points. Finally, 69 patients with PCS were included in the analysis.

Participants' characteristics

Anthropometric and clinical characteristic of patients are shown in Table 1. Women and men did differ for body weight, body fat, FEV_1, FEV_{ex} and the FAS score (Table 1). Pre-existing illnesses were cardiovascular disease (n = 1), depression (n = 6), diabetes (n = 5), hypertension (n = 15), COPD (n = 6), restrictive lung disease (n = 2), renal insufficiency (n = 1), and also 20 use tobacco. 21 of the patients have no pre-existing illnesses. Patients were taking antihypertensives (n = 14), ß-blockers (n = 6), antiobstructive medications (n = 16), antidepressants (n = 7), antidiabetics (n = 3), anticoagulants (n = 3), thyroid hormones (n = 11) and 25 patients were not taking any medications. Out of 69 patients two were not vaccinated, 30 once, 26 twice, and 11 thrice vaccinated against SARS-CoV-2 at study inclusion. Regarding vaccination status there were no differences between vaccination subgroups in physical performance, fatigue severity, and mental or physical component scores of HrQol.

Table 1. Subject characteristics at baseline.

	Total group	women	men	p-value
Subjects (women/men)	69	46	23	0.006
Age (years)	46 ± 12	46 ± 11	46 ± 12	0.820
Body weight (kg)	85.2 ± 22.4	79.2 ± 23.4	97.3 ± 14.4	<0.001
Body mass index (kg/m^2)	28.5 [11.1]	25.7 [12.1]	29.2 [9.0]	0.114
Body fat (%)	32.1 ± 11.6	35.2 ± 11.6	25.6 ± 8.6	<0.001
weeks since Covid-19 diagnosis	43 [32]	43 [30]	36 [42]	0.378
steps per day	7159 [3641]	6934 [3570]	7444 [3397]	0.445
maximum power output (watt/kg)	1.67 [0,90]	1.67 [0.95]	1.70 [0.80]	0.653
VO2max (ml/min/kg)	22.5 ± 6.4	21.9 ± 6.4	23.8 ± 6.3	0.248
6MWT (m)	525 ± 88	516 ± 83	543 ± 96	0.265
max HR ergometry (bpm)	150 ± 22	153 ± 21	145 ± 23	0.188
Breathing reserve (%)	39 [20]	37 [23]	43 [17]	0.408
FEV1 (ml)	3055 [970]	2910 [580]	3655 [985]	<0.001
FVCex (ml)	3983 ± 1043	3495 ± 776	4892 ± 857	<0.001
max Lactat concentration (mmol/l)	5.12 ± 2.19	5.12 ± 2.42	5.12 ± 1.67	0.995
Borg-scale	19 [1]	19 [1]	19 [1]	0.405
FAS score	35.0 ± 7.4	36.6 ± 6.7	32.0 ± 8.0	0.024
QoL physical score	35.2 ± 9.0	34.6 ± 8.9	36.4 ± 9.4	0.478
Qol mental score	40.9 ± 12.8	40.6 ± 13.9	41.6 ± 10.8	0.738
HADS depression score	6.0 [7.0]	5.0 [7.0]	8.0 [6.0]	0.279
HADS anxiety score	6.5 [8.0]	6.0 [9.0]	7.0 [7.0]	0.714

Differences between groups were analyzed with *Students t-test* for unpaired samples or the Mann-Whitney test, data are mean ± SD or median [interquartile range].

https://doi.org/10.1371/journal.pone.0292928.t001

Exercise capacity and health-related quality of life

The exercise capacity when expressed to age- and sex-specific norm values, was 72.3±18.5% predicted for $VO2_{max}$, 88.1±16.1% predicted for the 6-min walking distance, 99.6±18.7% predicted for FEV_1 and 104.1±18.5% predicted for FVC_{ex} (see also Fig 1 for individual data).

The mental component score and the physical component score for HrQoL were below norm values (79.4±24.9% predicted; 70.1±18.0% predicted, respectively) (for more detailed data see Figs 2 and 3 and Table 2 for parameters divided into fatigue and extreme fatigue).

Association of PCS severity and health-related outcomes

The FAS score was correlated to the 6MWT (r: -0.459, p<0.001), to the HrQoL physical component score (r: -0.366, p = 0.003), to the HrQoL mental score (r: -0.410, p<0.001), to the HADS depression score (r: 0.331, p = 0.009) and the HADS anxiety score (r: 0.290, p = 0.022). The FAS score was correlated only by trend to maximum power output (r:-0.225, p = 0.067) and to $VO2_{max}$ (r: -0.228, p = 0.064).

In a stepwise backward multivariate linear regression analysis with the FAS score as dependent variable and age, sex, time since diagnosis of PCS, $VO2_{max}$, 6MWT, HrQoL physical and mental component score, HADS depression and anxiety score as independent variables, sex (β = 0.220, p = 0.025), age (β = -0.236, p = 0.022), the HrQoL physical component score (β = –0.440, p<0.001), the HrQoL mental component score (β = –0.512, p<0.001) and by trend the 6MWT (β = –0.215, p = 0.068) predicted the FAS score. The model explained 53.2% of the total variation in PCS severity (FAS score).

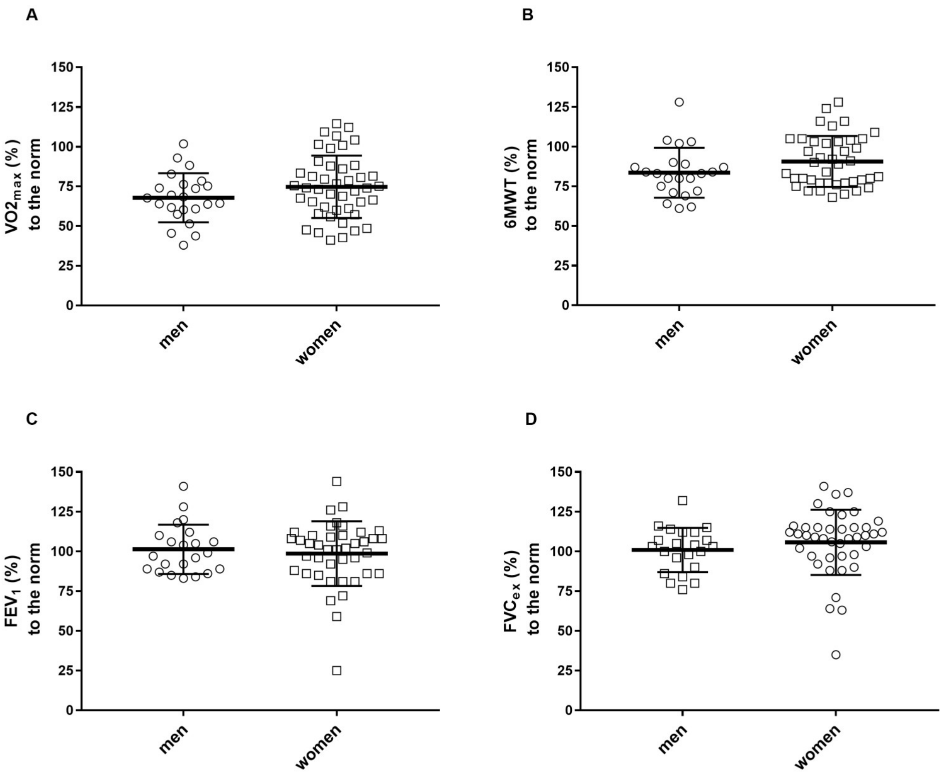

Fig 1. Exercise capacity (A: $VO2_{max}$; B: 6MWT) and pulmonary capacity (C: FEV_1; D: FVC_{ex}) in relation to the norm values. Data are individual values with mean ± SD.

https://doi.org/10.1371/journal.pone.0292928.g001

Discussion

We investigated anthropometric and clinical features of PCS patients and their association to PCS fatigue severity. Main finding of the study is that patients with PCS show reduced maximal and submaximal physical performance as well as limitations in quality of life, particularly pronounced in the physical components (especially in the physical role function). These results are essentially influenced by the severity of fatigue.

In addition to the risk factors mentioned above, obesity is also one of the predictors for the development of PCS [26], and BMI as well as body fat percentage were also elevated in our patient population. Obesity often is associated with a low cardiorespiratory fitness which also increases the risk for a severe course of COVID-19 as well as for the development of PCS [13].

As in other studies, our patient population showed a reduced $VO2_{max}$ as well as a reduced walking distance in the 6MWT [27–29]. The performance in percentage was more limited in the maximal ranges than in the submaximal range, although the FAS correlated significantly only with the 6MWT performance, a finding also observed in other studies [6], and tended to correlate with $VO2_{max}$. Possible causes of impaired cardiopulmonary fitness can be, in addition to reduced fitness even before the onset of the disease, a further reduction due to inactivity and a sedentary lifestyle because of COVID-19 related symptoms [29] or government-ordered measures of quarantine [30, 31]. Further reasons might include COVID-19-induced physical impairments, side effects of drug treatment or a combination of these factors [32].

The leading cause for discontinuing the exercise test in our patients was peripheral exhaustion (n = 45). Seeßle at al. [3] describe that about 40% of PCS patients still suffer from dyspnea

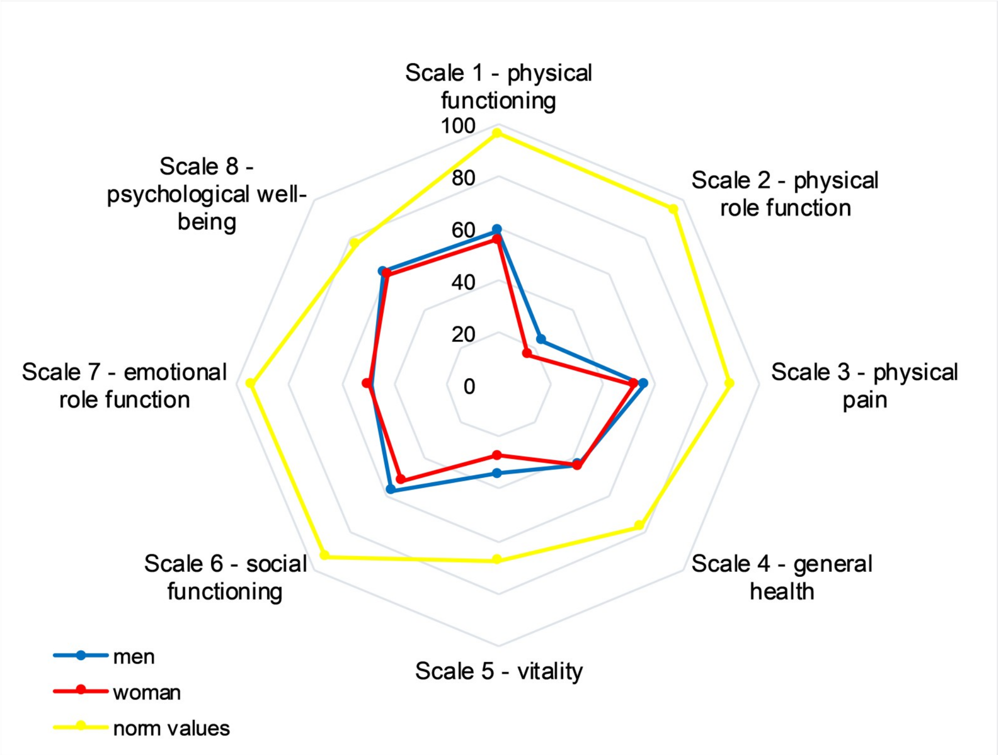

Fig 2. Subscales of the HrQoL questionnaire for men and women compared to the norm values.

https://doi.org/10.1371/journal.pone.0292928.g002

after about one year. In our study, this was not observed in this high number either at rest (Fig 1) or during spiroergometric exercise testing. During spiroergometry, we detected specific pulmonary limitations in two patients with chronic obstructive pulmonary disease and in two patients with restrictive breathing patterns. Although SARS-CoV-2 vaccination has been observed to reduce the risk of PCS, with two vaccinations appearing to be more effective than one [33] and the majority of our examined patients had been vaccinated at least once, the severity of PCS-related outcomes was not influenced by vaccination status in our cases.

Interestingly, although most of the patients subjectively reached a high level of self-perceived exertion at the BORG scale, in some cases there was no corresponding increase in heart rate, breathing rate and lactate concentration, which argues against cardiopulmonary or metabolic exhaustion in this context. Since this occurred predominantly in patients with a high FAS score we assume that central factors are partly limiting exercise capacity in these patients. In fact, mental fatigue could lead, due to increased cerebral adenosine activity amongst other factors, to a stronger perception of exertion during exercise [34]. This assumption is supported by Gibson et al. [35] who found no limitations in regard to muscle function in patients with chronic fatigue syndrome and demonstrated normal muscle physiology before exercise and during recovery. In addition these patients had increased perceived exertion scores during exercise in relation to heart rate and blood lactate concentration [35, 36], a finding similar to that in our PCS patients with high FAS scores. However, more recent research suggests that impaired muscle function could also contribute to decreased exercise capacity in patients with PCS [11, 37], which demonstrates the need for further research on this area.

Independent of COVID-19, post-infectious fatigue is a common phenomenon that can last even longer than 6 months after various infections (e.g. Epstein-Barr virus). Data on the

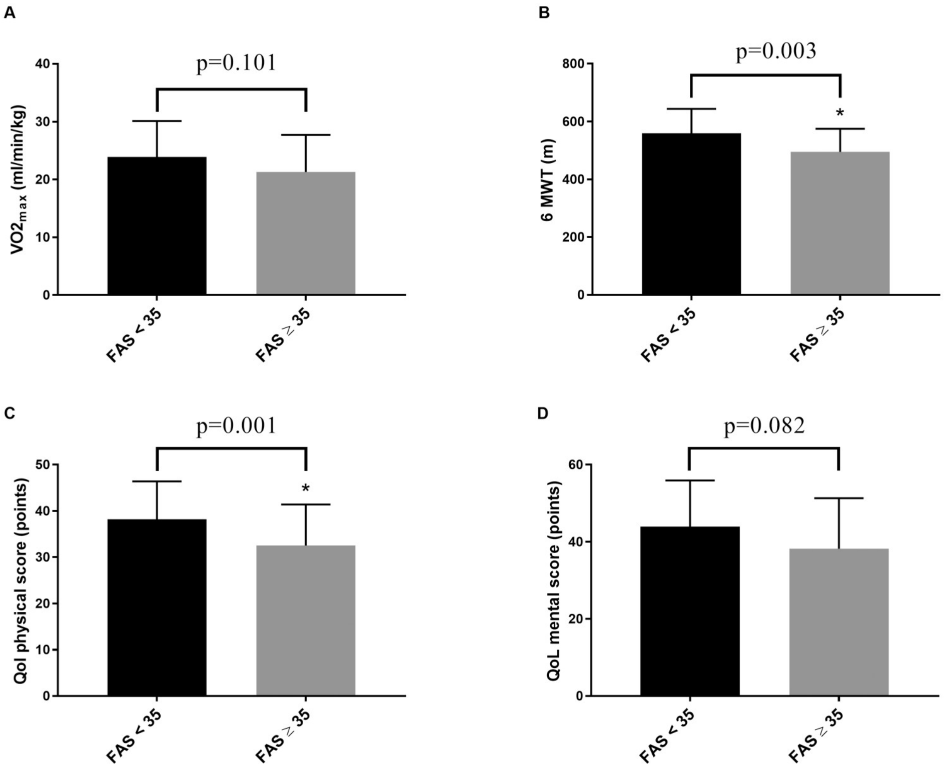

Fig 3. Exercise capacity (A: $VO2_{max}$; B: 6MWT) and HrQoL (C: Physical component score; D: Mental component score) divided into mild and severe fatigue based on FAS score. The framed p-values are given for differences between mild and severe fatigue as analyzed by Student t test for unpaired samples. Data are mean ± SD.

https://doi.org/10.1371/journal.pone.0292928.g003

incidence of fatigue after COVID-19 infection show that the number of affected patients varies widely, ranging up to 34% after hospitalization [38, 39]. Our study samples reflects the composition of the general population with and without pre-existing illness. Most of those illnesses in our study cohort are known for limiting quality of life and physical performance to certain extents (e.g. depressive symptoms, diabetes, hypertension). We cannot consider with certainty the degree of influence of the pre-existing illness on our observations regarding reduced physical performance and health-related quality of life relative to norm values. However, because

Table 2. Subscales of the HrQoL questionnaire for men and women compared to the norm values.

	men	women	men (% of the norm)	women (% of the norm)
Scale 1—physical functioning	59.1±21.0	55.5±21.0	61.4	57.7
Scale 2—physical role function	23.9±31.3	16.5±22.8	25.0	17.3
Scale 3—physical pain	56.1±25.0	52.3±25.6	63.1	58.9
Scale 4—general health	43.1±14.8	43.9±17.3	55.9	56.9
Scale 5—vitality	34.1±16.7	27.2±15.5	50.5	40.3
Scale 6—social functioning	57.4±32.0	51.8±27.0	61.6	55.7
Scale 7—emotional role function	48.5±45.7	49.6±44.8	51.3	52.5
Scale 8—psychological well-being	61.6±18.4	59.6±23.1	80.6	77.9

Data are mean ± SD.

https://doi.org/10.1371/journal.pone.0292928.t002

the reference values for these outcomes were also assessed from the general population, a major influence on study outcomes is unlikely.

Quality of life is known to be reduced in patients with PCS [29, 40]. However, there is controversy over which items are most affected. A systematic review in which 7 studies using the SF-36 questionnaire in PCS patients found lowered mean values for the total scores ranging from 52.7–79.96, whereas our result with 45.7 was clearly below the lower range. Indeed, an inclusion criteria for this study was a high FAS score, suggesting a pre-selection of our patients. While in five studies of the review [24] the subscale physical function (scale 1) had the best value, in three studies of the review in a recent cross-sectional study [41] and also in our study, the physical role (scale 2) was most affected. In contrast to us, Maes et al. [42] could not observe any impairment in the area of social HrQoL in PCS patients. A multicenter study that had investigated the influence of obesity on various PCS symptoms on average found somewhat lower values for depression and anxiety severity than we did [43], although our mean values were still within a normal range. Our observed associations between fatigue severity, physical performance and quality of life may be attributed to the interlinked nature of these parameters. In general, physical exercises leads to an improvement in physical well-being and mood. The lack of physical activity can be secondary to PCS-related fatigue, which in turn lead to a reduced formation of serotonin, endorphin, brain-derived neurotrophic factor, and vascular endothelial growth factor, leading to restricted neurogenesis, which in turn results in reduced neuronal plasticity with the consequence of increased depression, anxiety and reduced self-esteem [44]. From these interactions a vicious cycle can manifest, which makes a therapy of PCS even more complicated.

In the case of an assessment for returning to work not only the physical performance but also significantly reduced quality of life and fatigue should always be considered. In addition, health-related quality of life is closely linked with fatigue and other physio-somatic symptoms [39], therefore a multimodal therapy concept should be offered. Treatment therefore should be symptom-oriented and consider the required medical specialties e.g. pneumology, cardiology, neurology or psychosomatic medicine. Our data, that demonstrate the interlinkage of physical, mental and social aspects of health, supports the note that exercise should be one part within a multimodal treatment concept considering reduced mobility, breathlessness, stress, and problems with mood, memory, attention and tasks of daily living. The training program could be based on the WHO recommendations [45], taking into account a patient's individual resilience, mobility, and any other health condition. The WHO recommendation also points out that exercise should incorporate endurance, strength, but also balance and breathing techniques. Finally, overloading due to physical exercises training should be prevented. Thus, in a state of extreme fatigue, avoiding strenuous exercise is crucial to prevent post-exertional malaise or a crash [1]. According to the study of Davis et al. triggers for relapses can be inadequate exercise, physical or mental activity in relation to the extent of the disease as well as stress and after 7 months suffering from PCS more than half of the patients were found to be unable to work or only to a limited extent [46].

Our study has strengths and limitations. The strengths are that we investigated physical performance (by means of spiroergometry and on basis of the lactate performance curve) as well as quality of life and fatigue and their relationship to each other. The major limitation of the study is the relatively small sample number. This allows only results with high effect sizes to be recorded with sufficient accuracy and also limit the generalizability of the findings, particularly in such a heterogeneous sample of patients. Pre-COVID-19 state of health was not known and the extent of the deterioration thus not clearly definable. Furthermore, the study was performed in a German, mainly Caucasian population and, therefore, may not have sufficient generalizability to other nonwestern populations or ethnicities. Accordingly the reported

findings of our study should be interpreted with caution and further large-scale studies are needed to verify the observed results.

Conclusions

Patients with fatigue due to PCS show impaired physical performance and quality of life. These three items seem to be interdependent and influence each other. Our findings support the need for further investigations in regard to multimodal therapy concepts which should be adapted to the individual's performance in order to reduce fatigue and therefore to improve physical capacity and quality of life.

Acknowledgments

The results of this study are presented clearly, honestly, and without fabrication, falsification, or inappropriate data manipulation. We thank the patients for their study participation.

Author Contributions

Conceptualization: Meike Dirks, Uwe Tegtbur, Nora Drick, Arno Kerling.

Data curation: Sebastian Beyer, Nora Drick.

Formal analysis: Sebastian Beyer, Sven Haufe, Isabell Pink.

Funding acquisition: Uwe Tegtbur, Arno Kerling.

Investigation: Sebastian Beyer, Meike Dirks, Michèle Scharbau, Viktoria Lampe, Isabell Pink, Nora Drick, Arno Kerling.

Methodology: Sven Haufe, Alexandra Dopfer-Jablonka, Arno Kerling.

Project administration: Sebastian Beyer.

Resources: Alexandra Dopfer-Jablonka.

Supervision: Sven Haufe, Uwe Tegtbur, Arno Kerling.

Visualization: Sebastian Beyer.

Writing – original draft: Sebastian Beyer, Sven Haufe, Arno Kerling.

Writing – review & editing: Sebastian Beyer, Sven Haufe, Meike Dirks, Michèle Scharbau, Viktoria Lampe, Alexandra Dopfer-Jablonka, Uwe Tegtbur, Isabell Pink, Nora Drick, Arno Kerling.

References

1. Davis HE, McCorkell L, Vogel JM, Topol EJ. Long COVID: major findings, mechanisms and recommendations. Nat Rev Microbiol 2023 Mar; 21(3):133–146. https://doi.org/10.1038/s41579-022-00846-2 PMID: 36639608
2. Soriano JB, Murthy S, Marshall JC, Relan P, Diaz JV, WHO Clinical Case Definition Working Group on Post-COVID-19 Condition. A clinical case definition of post-COVID-19 condition by a Delphi consensus. Lancet Infect Dis 2022 Apr; 22(4):e102–e107. https://doi.org/10.1016/S1473-3099(21)00703-9 PMID: 34951953
3. Seeßle J, Waterboer T, Hippchen T, Simon J, Kirchner M, Lim A, et al. Persistent Symptoms in Adult Patients 1 Year After Coronavirus Disease 2019 (COVID-19): A Prospective Cohort Study. Clin Infect Dis 2022 Apr 9; 74(7):1191–1198. https://doi.org/10.1093/cid/ciab611 PMID: 34223884
4. Asadi-Pooya AA, Akbari A, Emami A, Lotfi M, Rostamihosseinkhani M, Nemati H, et al. Risk Factors Associated with Long COVID Syndrome: A Retrospective Study. Iran J Med Sci 2021 Nov; 46(6):428–436. https://doi.org/10.30476/ijms.2021.92080.2326 PMID: 34840383

5. Townsend L, Dowds J, O'Brien K, Sheill G, Dyer AH, O'Kelly B, et al. Persistent Poor Health after COVID-19 Is Not Associated with Respiratory Complications or Initial Disease Severity. Ann Am Thorac Soc 2021 Jun; 18(6):997–1003. https://doi.org/10.1513/AnnalsATS.202009-1175OC PMID: 33413026
6. Paradowska-Nowakowska E, Łoboda D, Gołba KS, Sarecka-Hujar B. Long COVID-19 Syndrome Severity According to Sex, Time from the Onset of the Disease, and Exercise Capacity-The Results of a Cross-Sectional Study. Life (Basel) 2023 Feb 11; 13(2):508. https://doi.org/10.3390/life13020508 PMID: 36836865
7. Crook H, Raza S, Nowell J, Young M, Edison P. Long covid-mechanisms, risk factors, and management. BMJ 2021 Jul 26; 374:n1648. https://doi.org/10.1136/bmj.n1648 PMID: 34312178
8. Jimeno-Almazán A, Martínez-Cava A, Buendía-Romero Á, Franco-López F, Sánchez-Agar JA, Sánchez-Alcaraz BJ, et al. Relationship between the severity of persistent symptoms, physical fitness, and cardiopulmonary function in post-COVID-19 condition. A population-based analysis. Intern Emerg Med 2022 Nov; 17(8):2199–2208. https://doi.org/10.1007/s11739-022-03039-0 PMID: 35904700
9. Warburton DER, Nicol CW, Bredin SSD. Health benefits of physical activity: the evidence. CMAJ 2006 Mar 14; 174(6):801–809. https://doi.org/10.1503/cmaj.051351 PMID: 16534088
10. Szekely Y, Lichter Y, Sadon S, Lupu L, Taieb P, Banai A, et al. Cardiorespiratory Abnormalities in Patients Recovering from Coronavirus Disease 2019. J Am Soc Echocardiogr 2021 Dec; 34(12):1273–1284.e9. https://doi.org/10.1016/j.echo.2021.08.022 PMID: 34508837
11. Durstenfeld MS, Sun K, Tahir P, Peluso MJ, Deeks SG, Aras MA, et al. Use of Cardiopulmonary Exercise Testing to Evaluate Long COVID-19 Symptoms in Adults: A Systematic Review and Meta-analysis. JAMA Netw Open 2022 Oct 3; 5(10):e2236057. https://doi.org/10.1001/jamanetworkopen.2022.36057 PMID: 36223120
12. Contreras AM, Newman DB, Cappelloni L, Niven AS, Mueller MR, Ganesh R, et al. Cardiopulmonary testing in long COVID-19 versus non-COVID-19 patients with undifferentiated Dyspnea on exertion. Prog Cardiovasc Dis 2023 May 19. https://doi.org/10.1016/j.pcad.2023.05.005 PMID: 37211198
13. Brawner CA, Ehrman JK, Bole S, Kerrigan DJ, Parikh SS, Lewis BK, et al. Inverse Relationship of Maximal Exercise Capacity to Hospitalization Secondary to Coronavirus Disease 2019. Mayo Clin Proc 2021 Jan; 96(1):32–39. https://doi.org/10.1016/j.mayocp.2020.10.003 PMID: 33413833
14. Ruegsegger GN, Booth FW. Health Benefits of Exercise. Cold Spring Harb Perspect Med 2018 Jul 2; 8 (7):a029694. https://doi.org/10.1101/cshperspect.a029694 PMID: 28507196
15. Dieli-Conwright CM, Courneya KS, Demark-Wahnefried W, Sami N, Lee K, Sweeney FC, et al. Aerobic and resistance exercise improves physical fitness, bone health, and quality of life in overweight and obese breast cancer survivors: a randomized controlled trial. Breast Cancer Res 2018 Oct 19; 20 (1):124–6. https://doi.org/10.1186/s13058-018-1051-6 PMID: 30340503
16. Lee BJ, Go JY, Kim AR, Chun SM, Park M, Yang DH, et al. Quality of Life and Physical Ability Changes After Hospital-Based Cardiac Rehabilitation in Patients With Myocardial Infarction. Annals of rehabilitation medicine 2017; 41(1):121–128. https://doi.org/10.5535/arm.2017.41.1.121 PMID: 28289644
17. Poudel AN, Zhu S, Cooper N, Roderick P, Alwan N, Tarrant C, et al. Impact of Covid-19 on health-related quality of life of patients: A structured review. PLoS One 2021 Oct 28; 16(10):e0259164. https://doi.org/10.1371/journal.pone.0259164 PMID: 34710173
18. Ferreira LN, Pereira LN, da Fé Brás M, Ilchuk K. Quality of life under the COVID-19 quarantine. Qual Life Res 2021 May; 30(5):1389–1405. https://doi.org/10.1007/s11136-020-02724-x PMID: 33389523
19. Cindrich SL, Lansing JE, Brower CS, McDowell CP, Herring MP, Meyer JD. Associations Between Change in Outside Time Pre- and Post-COVID-19 Public Health Restrictions and Mental Health: Brief Research Report. Front Public Health 2021 Jan 26; 9:619129. https://doi.org/10.3389/fpubh.2021.619129 PMID: 33585393
20. Borg G. Borg's perceived exertion and pain scales. Champaign, IL, US: Human Kinetics; 1998.
21. Löllgen H, Leyk D. Exercise Testing in Sports Medicine. Dtsch Arztebl Int 2018 Jun 15; 115(24):409–416. https://doi.org/10.3238/arztebl.2018.0409 PMID: 29968559
22. Rapp D, Scharhag J, Wagenpfeil S, Scholl J. Reference values for peak oxygen uptake: cross-sectional analysis of cycle ergometry-based cardiopulmonary exercise tests of 10 090 adult German volunteers from the Prevention First Registry. BMJ Open 2018 Mar 5; 8(3):e018697–018697.
23. ATS Committee on Proficiency Standards for Clinical Pulmonary Function Laboratories. ATS statement: guidelines for the six-minute walk test. Am J Respir Crit Care Med 2002 Jul 1; 166(1):111–117. https://doi.org/10.1164/ajrccm.166.1.at1102 PMID: 12091180
24. Nandasena H M R K G, Pathirathna ML, Atapattu A M M P, Prasanga PTS. Quality of life of COVID 19 patients after discharge: Systematic review. PLoS One 2022 Feb 16; 17(2):e0263941. https://doi.org/10.1371/journal.pone.0263941 PMID: 35171956

25. Snaith RP. The Hospital Anxiety And Depression Scale. Health Qual Life Outcomes 2003 Aug 1; 1:29–29. https://doi.org/10.1186/1477-7525-1-29 PMID: 12914662

26. Subramanian A, Nirantharakumar K, Hughes S, Myles P, Williams T, Gokhale KM, et al. Symptoms and risk factors for long COVID in non-hospitalized adults. Nat Med 2022 Aug; 28(8):1706–1714. https://doi.org/10.1038/s41591-022-01909-w PMID: 35879616

27. Clavario P, De Marzo V, Lotti R, Barbara C, Porcile A, Russo C, et al. Cardiopulmonary exercise testing in COVID-19 patients at 3 months follow-up. Int J Cardiol 2021 Oct 1; 340:113–118.

28. Rinaldo RF, Mondoni M, Parazzini EM, Pitari F, Brambilla E, Luraschi S, et al. Deconditioning as main mechanism of impaired exercise response in COVID-19 survivors. Eur Respir J 2021 Aug 26; 58 (2):2100870. Print 2021 Aug. https://doi.org/10.1183/13993003.00870-2021 PMID: 33926969

29. Sirayder U, Inal-Ince D, Kepenek-Varol B, Acik C. Long-Term Characteristics of Severe COVID-19: Respiratory Function, Functional Capacity, and Quality of Life. Int J Environ Res Public Health 2022 May 23; 19(10):6304. https://doi.org/10.3390/ijerph19106304 PMID: 35627841

30. Hariri N, Takrooni W, Nasraldin N, Bawahab N, Alfalogy E. Effect of the Long COVID-19 Quarantine and Associated Lack of Physical Activity on Overall Health. Cureus 2022 Nov 1; 14(11):e30955. https://doi.org/10.7759/cureus.30955 PMID: 36465224

31. Wu Z, Wang Y, Zhang Y, Bennell KL, White DK, Shen L, et al. The COVID-19 Pandemic and Daily Steps in the General Population: Meta-analysis of Observational Studies. JMIR Public Health Surveill 2023 May 30; 9:e40650. https://doi.org/10.2196/40650 PMID: 37252779

32. Schwendinger F, Knaier R, Radtke T, Schmidt-Trucksäss A. Low Cardiorespiratory Fitness Post-COVID-19: A Narrative Review. Sports Med 2023 Jan; 53(1):51–74. https://doi.org/10.1007/s40279-022-01751-7 PMID: 36115933

33. Byambasuren O, Stehlik P, Clark J, Alcorn K, Glasziou P. Effect of covid-19 vaccination on long covid: systematic review. BMJ Med 2023 Feb 1; 2(1):e000385–000385. eCollection 2023. https://doi.org/10.1136/bmjmed-2022-000385 PMID: 36936268

34. Martin K, Meeusen R, Thompson KG, Keegan R, Rattray B. Mental Fatigue Impairs Endurance Performance: A Physiological Explanation. Sports Med 2018 Sep; 48(9):2041–2051. https://doi.org/10.1007/s40279-018-0946-9 PMID: 29923147

35. Gibson H, Carroll N, Clague JE, Edwards RH. Exercise performance and fatiguability in patients with chronic fatigue syndrome. J Neurol Neurosurg Psychiatry 1993 Sep; 56(9):993–998. https://doi.org/10.1136/jnnp.56.9.993 PMID: 8410041

36. Vollrath S, Matits L, Schellenberg J, Kirsten J, Steinacker JM, Bizjak DA. Decreased physical performance despite objective and subjective maximal exhaustion in post-COVID-19 individuals with fatigue. Eur J Med Res 2023 Aug 26; 28(1):298–5. https://doi.org/10.1186/s40001-023-01274-5 PMID: 37633931

37. Colosio M, Brocca L, Gatti M, Neri M, Crea E, Cadile F, et al. Structural and functional impairments of skeletal muscle in patients with post-acute sequelae of SARS-CoV-2 infection. J Appl Physiol (1985) 2023 Sep 7.

38. Pilotto A, Cristillo V, Cotti Piccinelli S, Zoppi N, Bonzi G, Sattin D, et al. Long-term neurological manifestations of COVID-19: prevalence and predictive factors. Neurol Sci 2021 Dec; 42(12):4903–4907. https://doi.org/10.1007/s10072-021-05586-4 PMID: 34523082

39. Sandler CX, Wyller VBB, Moss-Morris R, Buchwald D, Crawley E, Hautvast J, et al. Long COVID and Post-infective Fatigue Syndrome: A Review. Open Forum Infect Dis 2021 Sep 9; 8(10):ofab440. https://doi.org/10.1093/ofid/ofab440 PMID: 34631916

40. Mastrorosa I, Del Duca G, Pinnetti C, Lorenzini P, Vergori A, Brita AC, et al. What is the impact of post-COVID-19 syndrome on health-related quality of life and associated factors: a cross-sectional analysis. Health Qual Life Outcomes 2023 Mar 22; 21(1):28–z. https://doi.org/10.1186/s12955-023-02107-z PMID: 36949439

41. AlRasheed MM, Al-Aqeel S, Aboheimed GI, AlRasheed NM, Abanmy NO, Alhamid GA, et al. Quality of Life, Fatigue, and Physical Symptoms Post-COVID-19 Condition: A Cross-Sectional Comparative Study. Healthcare (Basel) 2023 Jun 5; 11(11):1660. https://doi.org/10.3390/healthcare11111660 PMID: 37297800

42. Maes M, Al-Rubaye HT, Almulla AF, Al-Hadrawi DS, Stoyanova K, Kubera M, et al. Lowered Quality of Life in Long COVID Is Predicted by Affective Symptoms, Chronic Fatigue Syndrome, Inflammation and Neuroimmunotoxic Pathways. Int J Environ Res Public Health 2022 Aug 19; 19(16):10362. https://doi.org/10.3390/ijerph191610362 PMID: 36011997

43. Fernández-de-Las-Peñas C, Torres-Macho J, Elvira-Martínez CM, Molina-Trigueros LJ, Sebastián-Viana T, Hernández-Barrera V. Obesity is associated with a greater number of long-term post-COVID symptoms and poor sleep quality: A multicentre case-control study. Int J Clin Pract 2021 Dec; 75(12): e14917. https://doi.org/10.1111/ijcp.14917 PMID: 34569684

44. Schulz K, Meyer A, Langguth N. Exercise and psychological well-being. Bundesgesundheitsblatt Gesundheitsforschung Gesundheitsschutz 2012 Jan; 55(1):55–65.

45. World Health Organization. Support for Rehabilitation: Self-Management after COVID-19 Related Illness. 2021; https://apps.who.int/iris/handle/10665/344472. Accessed 22.09.2023.

46. Davis HE, Assaf GS, McCorkell L, Wei H, Low RJ, Re'em Y, et al. Characterizing long COVID in an international cohort: 7 months of symptoms and their impact. EClinicalMedicine 2021 Aug; 38:101019. https://doi.org/10.1016/j.eclinm.2021.101019 PMID: 34308300
